# Mammalian Target of Rapamycin Inhibitors Induce Tumor Cell Apoptosis *In Vivo* Primarily by Inhibiting VEGF Expression and Angiogenesis

**DOI:** 10.1155/2013/897025

**Published:** 2013-02-28

**Authors:** Patrick Frost, Eileen Berlanger, Veena Mysore, Bao Hoang, YiJiang Shi, Joseph Gera, Alan Lichtenstein

**Affiliations:** ^1^Department of Hematology-Oncology, West Los Angeles VA Medical Center, Los Angeles, CA 90073, USA; ^2^Department of Medicine, The Jonsson Comprehensive Cancer Center, University of California, Los Angeles, CA 90095, USA

## Abstract

We found that rapalog mTOR inhibitors induce G1 arrest in the PTEN-null HS Sultan B-cell lymphoma line *in vitro*, but that administration of rapalogs in a HS Sultan xenograft model resulted in significant apoptosis, and that this correlated with induction of hypoxia and inhibition of neoangiogenesis and VEGF expression. Mechanistically, rapalogs prevent cap-dependent translation, but studies have shown that cap-independent, internal ribosome entry site (IRES)-mediated translation of genes, such as c-myc and cyclin D, can provide a fail-safe mechanism that regulates tumor survival. Therefore, we tested if IRES-dependent expression of VEGF could likewise regulate sensitivity of tumor cells *in vivo*. To achieve this, we developed isogenic HS Sultan cell lines that ectopically express the VEGF ORF fused to the p27 IRES, an IRES sequence that is insensitive to AKT-mediated inhibition of IRES activity and effective in PTEN-null tumors. Mice challenged with p27-VEGF transfected tumor cells were more resistant to the antiangiogenic and apoptotic effects of the rapalog, temsirolimus, and active site mTOR inhibitor, pp242. Our results confirm the critical role of VEGF expression in tumors during treatment with mTOR inhibitors and underscore the importance of IRES activity as a resistance mechanism to such targeted therapy.

## 1. Introduction

Whilst inhibitors of the mTOR signaling pathways have been approved for use against advanced renal cell carcinoma [1, 2], their effectiveness against hematological malignancies remains unclear [3]. Several rapalog mTOR inhibitors, including rapamycin [4, 5], temsirolimus (CCI-779) [6–9], and everolimus (RAD001) [10], have shown preclinical potential in hematological malignancies. However, one factor potentially limiting the effectiveness of rapalogs for treating hematological malignancies is the fact that *in vitro* exposure to mTOR inhibitors often only induces G1/S cell cycle arrest without apoptosis [11, 12]. Still, there is no doubt that, in some *in vivo* treated models, rapalogs can cause tumor cell death. Good clinical examples of this were seen in patients with mantle cell lymphoma [13] or nonmantle cell non-Hodgkin's lymphoma subtypes [14] treated with temsirolimus: objective responses were observed in some patients with reduction of tumor size. Thus, lack of *in vitro* tumor cell apoptosis may not accurately reflect the *in vivo* situation where tumor cell survival can be regulated by the microenvironment which itself may be impacted by mTOR inhibitors. 

In a prior report [6], we identified tumor cell apoptosis in mice treated with temsirolimus, while *in vitro* exposure only resulted in cytostasis. Subsequent studies [9] showed that *in vivo* apoptosis correlated with a downregulation of tumor VEGF expression and decrease in neoangiogenesis, suggesting that *in vivo* tumor cytotoxicity is due to indirect effects of the drugs that may result in increased oxygen and nutrient stress in the tumor microenvironment rather than direct drug-mediated apoptosis. Thus, we initiated the current study to definitively ask if altered VEGF expression and downregulation of angiogenesis were the key effects of mTOR inhibitors that mediated tumor cell apoptosis and tumor rejection *in vivo*. To do this we exploited the ability of a potent internal ribosome entry site (IRES) sequence [15] to allow ectopic cap-independent VEGF expression in xenograft tumors of mice treated with mTOR inhibitors. Our results confirm that the inhibition of VEGF translation induced by mTOR inhibitors participates in the observed tumor cell apoptosis and antitumor response *in vivo* and underscore the importance of IRES-mediated VEGF translation as a possible resistance mechanism to rapalogs.

## 2. Materials and Methods

### 2.1. Cell Lines

Cell lines were purchased from ATCC and maintained at 37°C and 5% CO_2_.

### 2.2. Reagents

Rapamycin was purchased from Calbiochem (San Diego, CA, USA). Temsirolimus (CCI-779) was provided by Wyeth-Ayerst (Pearl River, NY, USA) and was prepared as previously described [6]. Enzyme-linked immunosorbent assay (ELISA) kits specific for human VEGF was purchased from R and D Systems (Minneapolis, MN, USA), and the anti-FLAG coated 96-well plates were purchased from Sigma. The hypoxiprobe-1 kit was purchased from HPI Inc (Burlington, MA, USA).

### 2.3. Cell Cycle and Apoptosis Analysis

Cell cycle analysis of hypotonic propidium iodide- (PI-) stained cells was determined by fluorescence-activated cell sorting (FACS) with a Becton-Dickinson FACScaliber. Histograms generated by FACS were analyzed by ModFit Cell Cycle Analysis Software (Verity, Topsham, ME) to determine the percentage of cells in each phase. Cellular apoptosis was measured by FACS analysis using a kit for cleaved caspase-3 (Becton-Dickinson). 

### 2.4. Ectopic VEGF Construct

The 5'UTR of p27^kip1^ that contains a known 365 nucleotide IRES sequence [16, 17], was fused downstream of a FLAG tagged VEGF165 isoform open reading frame (ORF). The p27IRES-VEGF-Flag construct was subcloned into the pGL4.5 vector (Promega). This p27IRES fusion construct was previously shown to be capable of cap-independent translation of cyclin D1 and c-myc proteins in cells treated with rapalogs [15]. The p27IRES cloned in the opposite orientation to generate the (Rev)p27IRES-VEGF-Flag construct was used as a negative control.

### 2.5. Generation of Isogenic Cell Lines

Stably transfected HS Sultan cells were developed by transfecting cells using the AMAXA Nucleofection System (AMAXA Inc, Gaithersburg, MD) followed by selection with hygromycin (350 mg/mL) for 5–7 days. The transfection efficiency was typically >80% as determined by transfection of cells with a green fluorescent protein plasmid vector. Successful stable transfections of HS Sultan cell lines were determined by PCR using probes specific for either the p27IRES-VEGF or (Rev)p27IRES-VEGF-Flag construct. 

### 2.6. Animals

Male NOD/SCID mice (4–6 weeks old) were obtained from Jackson Laboratories (Bar Harbor, ME, USA). All animal studies were conducted in accordance with protocols approved by the Animal Research Committee of the West Los Angeles Veterans Administration Medical Center.

### 2.7. Xenograft Model

We used the murine myeloma xenograft model of LeBlanc et al. [18] with minor modifications [6]. The cell lines (1 × 10^6^ cells/mouse) were mixed with matrigel and were then injected subcutaneously into the flank (200 *μ*L/mouse) of the mice. Tumor growth was monitored daily, and mice were randomized into drug treated or control groups (8–14 mice/group) when the tumor volume reached approximately 200–400 mm^3^. The temsirolimus drug solution was prepared as a 50 mg/mL stock solution in 100% EtOH. On the day of injections, the drug was diluted in 5% Tween-80, 5% polyethylene glycol-400 (Sigma, St. Louis MO) to the appropriate final concentration (final concentration of EtOH is 0.4%). Temsirolimus (200 *μ*L) was administered IP daily × 5, followed by 2 days of no drug and then 5 additional daily injections (total of 10 injections) [19]. Mice were routinely euthanized when tumors reached >2000 mm^3^ in volume.

### 2.8. Immunohistochemistry

At day +13 (24 hours after last injection) some mice were euthanized with CO_2_, and the tumor mass was excised. The tumor was bisected using a razor blade: one-half of the tumor was immediately placed in 10% buffered formaldehyde overnight, and the other half was frozen for protein extraction. Formaldehyde fixed tumors were embedded in paraffin and cut into 5 *μ*m-thick serial sections using standard histological procedures. Immunohistochemical staining with anti-mouse CD-34 antibody (to measure microvessel density), cleaved caspase-3 (to measure apoptosis), and hypoxiprobe (HPI Inc) (to measure hypoxia) was conducted using standardized automated methods. 

### 2.9. Morphometric Analysis

IHC analysis was performed on tissue sections with a Nikon Microphot-SA microscope (Melville, NY) equipped with plan apochromat lenses (20X and 40X). A diagnostic technologies digital camera, model SPOT-RT, was used to capture images with a resolution of 1520 × 1080 pixels. MetaMorph software (version 6.1) (Universal Imaging Corporation, West Chester, PA) was used to measure percent of hypoxia stained tumor sections. Final images for publication were prepared using Adobe Photoshop software (version 7.0).

### 2.10. Statistics

Student's *t*-test was used to determine significance of differences between groups. Regression analysis was used to test for statistical differences in the slopes of the growth curve of tumor xenografts. Unless otherwise specified, data is presented as mean ± SEM. 

## 3. Results

### 3.1. Rapalogs Induce Tumor Cell Apoptosis *In Vivo* but Not *In Vitro*

**Figure 1:**
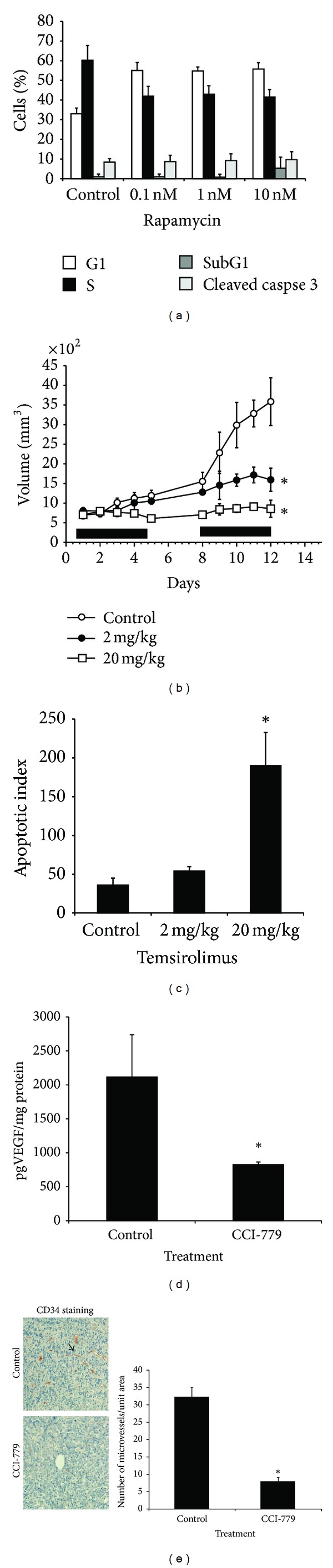
Antitumor effects on HS Sultan cells *in vivo* and *in vitro*. (a) Cell cycle and apoptosis analysis of HS Sultan cells. G1/S arrest induced by 48-hour incubation with rapamycin was measured by propidium iodide to determine the percentage of cells in each phase (G1, S, or sub-G1), and anticleaved caspase-3 antibody was used to determine the percentage of cells undergoing apoptosis. Results shown are mean ± SEM of 4 independent experiments. (b) NOD/SCID mice (6 mice/group) were challenged subcutaneously with HS Sultan cells. When tumor size reached approximately 500 mm^3^, mice were randomly assigned to receive vehicle alone or varying doses of temsirolimus IP for 10 days, as described in “Materials and Methods.” Results are presented as tumor volume (mean ± SEM). Solid bars on *x*-axis denote days of IP treatment. *Asterisks denote significant difference* (*P* < 0.05) *between control and temsirolimus-treated mice*. (c) Cleaved caspase-3 staining of HS Sultan xenografts harvested from mice at day 13 was used to identify apoptosis. Results are presented as number of cleaved caspase-3 stained cells/microscopic field (original magnification 20X), mean ± SD, *n* = 10 fields for each tumor, and 4 tumors/group. Asterisk denotes significant difference (*P* < 0.05) between control and temsirolimus-treated mice. (d) VEGF-specific ELISA assay for expression of VEGF collected from HS Sultan xenografts lysate harvested from mice treated with vehicle control or temsirolimus (tumors collected on day 13). Lysate was pooled (*N* = 4 tumor/group) and are presented as the mean ± STD. Asterisk denotes significant difference (*P* < 0.05) between control and temsirolimus-treated mice. (e) Left panel. Representative slides of CD34-stained HS Sultan xenograft sections from mice treated with vehicle control (top panel) or 20 mg/kg temsirolimus (bottom panel) harvested on day 13. Original magnification, ×40. Arrow shows microvessel. Right panel. Results represent number of microvessel/area of microscopic field (original magnification, ×20) stained positive for CD34 and assessed as described in “Materials and Methods.” Data are mean ± SD, *n* = 10 fields/tumor, and 4 tumors/group. Asterisk denotes significant difference (*P* < 0.05) between control and temsirolimus-treated mice.

In our previous studies, we demonstrated that 10 daily IP injections of temsirolimus had an antitumor effect in mice challenged with the human B-cell myeloma cell lines 8226, U226, and OPM-2 [6, 9, 20]. Furthermore, there was a significant correlation between the *in vivo* sensitivity of these cell lines to temsirolimus and their AKT activity. Specifically, the PTEN-null OPM-2 cell line with heightened basal AKT activation levels was the most sensitive to treatment with temsirolimus [6]. This was consistent with the previously published observations that PTEN-null or AKT hyperactive cells are more sensitive to rapalogs [12, 15, 19] and was confirmed using isogenic U266 cells stably transfected with a constitutively activated AKT allele [9, 20]. To further evaluate this relationship in the current study, we utilized the HS Sultan B-cell line that is also PTEN-null, expresses enhanced AKT activity, and is known to be hypersensitive to rapalogs [12]. As was previously reported for myeloma cell lines [12] and other B-cell malignancies [21], we found that *in vitro* treatment of HS Sultan cells with rapamycin was primarily cytostatic, inducing G1/S cell cycle arrest, rather than apoptotic (Figure 1(a)). We observed that the G1/S arrest was maximal even at as low a concentration of 0.1 nM with an increase in G1 distribution from 33% to 56% and a corresponding decrease in S phase from 60% to 41% (mean of 3 independent experiments). By assessment of sub-G1 peak distributions (Figure 1(a)), we found that there was not significant apoptosis detected in any of the experimental groups. In additional experiments assaying apoptosis using activated caspase-3 expression, similar results were observed (Figure 1(a)). The results in Figure 1(a) are from 48-hour incubations, but no apoptosis induction was likewise seen with longer incubations (72 hours, data not shown). Next, to test for a possible apoptotic effect* in vivo*, NOD/SCID mice were challenged SQ with HS Sultan cells, and once a palpable tumor was detected the mice were treated with temsirolimus as previously described with 5 daily IP injections, 2 days of rest, and 5 additional injections [6]. The change in tumor volume was measured every other day, and at the end of the drug regimen, the mice were sacrificed and tumors immediately collected for analysis. As shown in Figure 1(b), *in vivo* growth of HS Sultan tumors was extremely sensitive to temsirolimus in a dose dependent manner. In order to determine if tumor regression was due to apoptosis, sections from tumor harvested after the last treatment with temsirolimus were stained for cleaved caspase-3 and demonstrated a dose-dependent induction of apoptosis (determined by the number of apoptotic cells/unit area) (Figure 1(c)). Although 2 mg/kg was ineffective, a temsirolimus dose of 20 mg/kg increased detected apoptosis staining by >4-fold. In our previous studies using myeloma cell lines [9], we also showed that induction of tumor cell apoptosis in rapalog-treated mice correlated with decreased VEGF expression.  Figure 1(d) demonstrates that, in a similar fashion, tumor apoptosis in HS Sultan-challenged mice treated with 20 mg/kg temsirolimus also correlates with a significant (*P* < 0.05) inhibition of VEGF expression in the tumor lysates. This decrease in VEGF also correlated with a marked inhibition (*P* < 0.05) of microvessel density (MVD) in the tumor sections (Figure 1(e)). Representative sections stained for CD34 in tumor nodules from mice treated with vehicle control or 20 mg/kg temsirolimus are shown in the right panel (arrow indicates microvessel). Quantification of the decrease MVD in temsirolimus-treated mice is shown in Figure 1(e), left panel.

**Figure 2:**
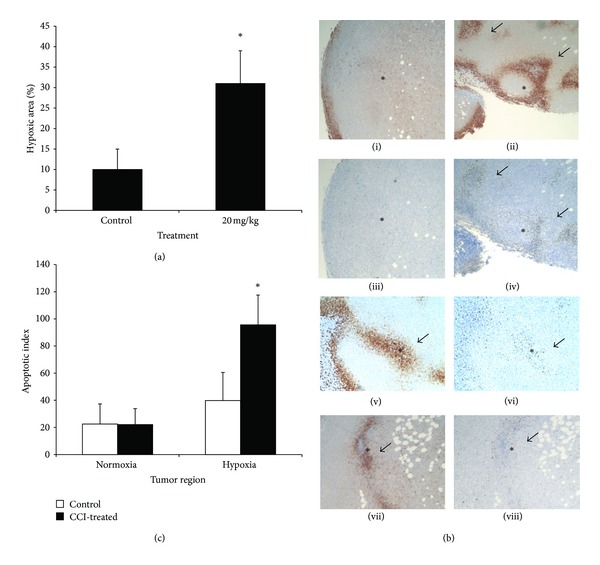
mTOR inhibition induces apoptosis and hypoxia in HS Sultan xenografts. (a) Analysis of pimonidazole staining (a marker of hypoxia) of HS Sultan xenografts harvested from mice at day 13. The area of pimonidazole staining/microscopic field (original magnification 20X) was measured by morphometric analysis as described in the “Materials and Methods.” Data are presented as mean ± SD, *n* = 10 fields for each tumor, and 4 tumors/group. Asterisk denotes significant difference (*P* < 0.05) between control and temsirolimus-treated mice. (b) Representative photomicrographs of immunohistochemistry of serial tumor sections of control or temsirolimus-treated tumors stained for hypoxia or cleaved caspase-3. *indicates same geographical location of tumor section pairs. Arrows indicate regions of apoptosis (in panels (ii) and (iv)) that colocalize to regions of hypoxia in corresponding serial section. Panels (i) (hypoxia) and (iii) (cleaved caspase-3) show serial sections from HS Sultan xenograft harvested on day 13 from vehicle control treated mouse. Panel (ii) (hypoxia) and panel (iv) (cleaved caspase-3) show serial sections from HS Sultan xenograft harvested on day 13 from 20 mg/kg temsirolimus-treated mouse. Additional serial tumor sections are shown in panel (v) (stained for hypoxia), panel (vi) (stained for apoptosis), panel (vii) (stained for hypoxia), and panel (viii) (stained for apoptosis) from HS Sultan xenografts harvested on day 13 from 20 mg/kg temsirolimus-treated mice. Arrows and (∗) in paired sections (panels (v) and (vi), panels (vii) and (viii)) indicate corresponding geographical regions. (c) Cleaved caspase-3 staining of HS Sultan xenografts harvested from mice at day 13 was used to identify apoptosis in normoxic and hypoxic regions. Hypoxic and normoxic regions were identified in pimonidazole-stained sections, and then the apoptotic index was determined in corresponding serial sections of cleaved caspase-3 stained slides. Results are number of cleaved caspase-3 stained cells/microscopic field (original magnification 20X) in either normoxic or hypoxic regions, mean ± SD, *n* = 10 fields for each tumor, and 4 tumors/group. Asterisk denotes significant difference (*P* < 0.05) between control and temsirolimus-treated mice.

To gain support for the notion that the temsirolimus-mediated inhibition of angiogenesis increased hypoxic stress and this was causally related to the induction of apoptosis in the tumor nodules, we tested whether tumor cell apoptosis was colocalized to regions of hypoxia. NOD/SCID mice were challenged with Sultan cells and treated with temsirolimus at 20 mg/kg or no treatment as described previously and then sacrificed one day after the last injection of temsirolimus. One hour before the tumors were harvested, the mice were given an IP injection of hypoxiprobe-1 (NPI) to identify regions of hypoxia in the tumor beds. The tumors were sectioned, and immunohistochemistry for pimonidazole staining was performed followed by morphometric analysis to assay for the total area of hypoxic regions in control and temsirolimus-treated mice. Low power microscopic analysis of randomly selected fields was performed by two of the authors (PF and AL) who were blinded to the treatments. The area of tumor hypoxia (determined by brown staining) that was measured in tumor sections (5 tumors/group, 5 fields/tumor) is summarized in Figure 2(a). Our results demonstrated that the pimonidazole-stained area of the tumor was observed in approximately 30% of the tumor section from tumor nodules harvested from the drug-treated mice, whereas only about 10% of the tumor sections were stained for hypoxia in tumor nodules harvested from mice treated with vehicle control. In addition, the pattern of hypoxia was remarkably different. Specifically, we observed that hypoxic regions in sections from tumors harvested from control mice were generally diffuse and lightly stained (Figure 2(b) panel (i)), whereas hypoxic regions in temsirolimus-treated mice tended to be more focused to discreet regions of the tumor nodule (as indicated by the brown stained areas (Figure 2(b) panel (ii))). 

In order to determine if there was a relationship between apoptosis and hypoxia, serial tumor sections were also stained for cleaved caspase-3. We observed that apoptotic tumor cells were primarily located within the regions of hypoxia in the temsirolimus-treated tumors (Figure 2(b) panel (iv)—arrows indicate regions of apoptosis to be compared to panel (ii), which is stained for hypoxia), whereas in control tumors, the distribution of apoptotic nuclei was randomly distributed within normoxic and hypoxic regions (Figure 2(b) panel (iii)). Additional serial tumor sections are shown for temsirolimus-treated mice stained for hypoxia (Figure 2(b) panels (v) and (vii)) and corresponding sections stained for caspase-3 (panels (vi) and (viii). Arrows indicate regions of apoptosis, and * indicates same geographical location in section pairs; panels (v) and (vi) and panels (vii) and (viii)). The geographical location of hypoxia and apoptosis was remarkably correlated in temsirolimus-treated tumors.

We next measured the apoptotic index (number of apoptotic cells/unit area) in normoxic or hypoxic regions of the tumor (Figure 2(c)). Briefly, under low power (20X), hypoxic (determined by brown staining) and “normoxic” (determined by a lack of staining) regions of the tumors were identified by microscopy. The number of apoptotic tumor cells in serial sections of the corresponding areas stained for cleaved caspase-3 was then counted (5 tumors/group, 5 fields/region) under higher power (40X). As shown in Figure 2(c), we observed an approximately 2-fold increase in apoptotic cells in the hypoxic regions of tumors from the drug-treated mice compared to hypoxic regions of the control tumors (*P* < 0.05). In contrast, there was little difference in the number of apoptotic nuclei located within normoxic regions of the tumor in either the control or temsirolimus-treated mice. 

### 3.2. P27 IRES-Mediated Expression of VEGF Can Overcome Sensitivity of VEGF Expression to Rapamycin *In Vitro*

**Figure 3:**
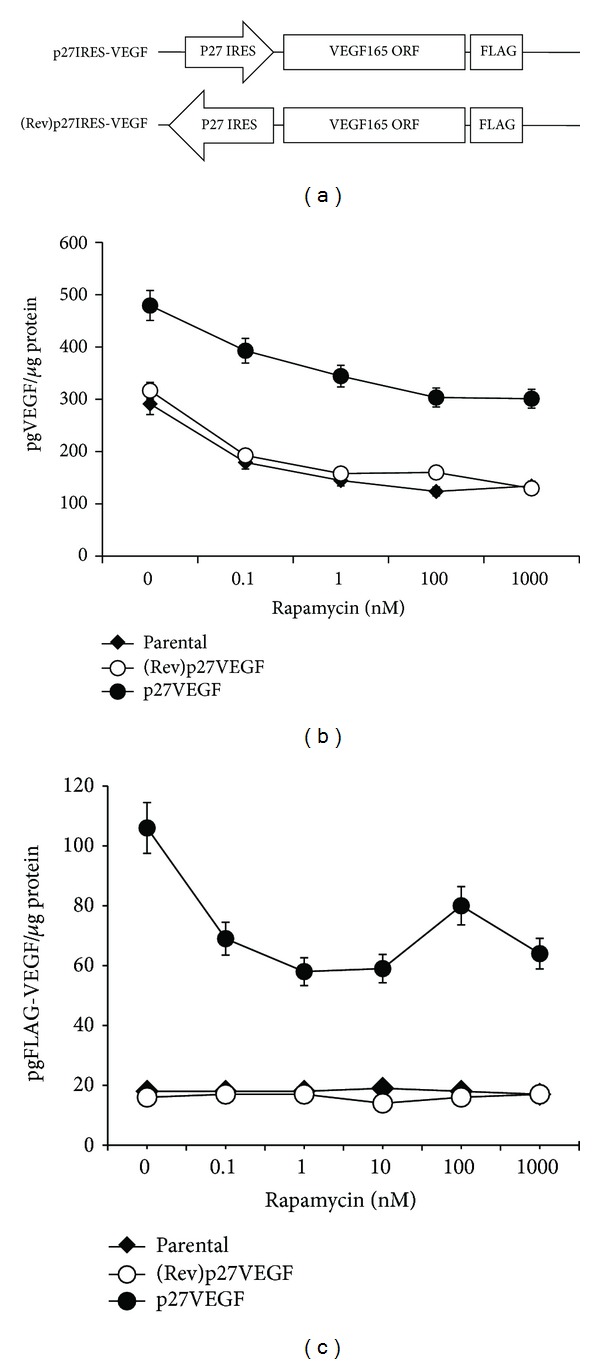
Characterization of exogenous VEGF. (a) Cartoon of plasmids used to generate exogenous VEGF. The p27 IRES was cloned upstream of the VEGF ORF. (b) Stably transfected isogenic HS Sultan cell lines or HS Sultan parental cell line were treated with indicated concentration of rapamycin for 48 hours. ELISA was used to determine the VEGF levels/mg of protein in cell supernatants in triplicate (data are presented as mean ± SD). (c) The levels of FLAG-tagged VEGF were measured in cellular supernatants (in triplicate) using anti-FLAG ELISA and anti-VEGF secondary antibody (data are presented as mean ± SD).

In our previous experiments, we showed that sensitivity to mTOR inhibition *in vivo* correlated with tumor VEGF expression [9]. In those experiments, we demonstrated that in rapalog-sensitive, AKT-activated tumors, VEGF expression was downregulated by drugs, but in rapalog-resistant, low AKT tumors, VEGF expression was maintained during drug treatment. Differential effects occurred at the level of VEGF translation; that is, RNA levels were comparable, but inhibition of VEGF protein expression only occurred in sensitive tumors. Further study confirmed that, during rapalog-induced inhibition of cap dependent VEGF translation, IRES-dependent VEGF translation was a fail-safe mechanism for VEGF expression and AKT activity curtailed IRES activity, thus explaining the AKT-dependent sensitivity. The heightened AKT activity in the PTEN-null HS Sultan tumors prevented IRES function and the fail-safe mechanisms of VEGF translation. Thus, to test if ectopic VEGF expression could rescue tumors from the apoptotic effects of rapalogs *in vivo*, we would need to express the VEGF transgene in such a way as to allow its IRES-dependent translation and expression, since rapalog treatment would significantly prevent its normal cap-dependent translation. To do this, we exploited the finding that the p27 IRES structure was not inhibited in its activity by heightened AKT activity [15]. Thus, the VEGF ORF was fused to the 5'UTR of p27^kip1^ that contains its 365 nucleotide IRES sequence [16, 17, 22]. The p27IRES sequence in a reverse orientation upstream of the VEGF ORF was used as a negative control (Figure 3(a)). Isogenic HS Sultan cell lines were generated by stably transfecting the cells with either p27IRES-VEGF or (Rev)p27IRES-VEGF control constructs, and faithful transcription of our fusion genes was determined by RT-PCR (data not shown). 

As shown in Figure 3(b), p27-IRES-VEGF expressing cells demonstrated significantly greater levels of VEGF than the parental and control-transfected cell lines, and these cells were relatively resistant to rapamycin-mediated inhibition of VEGF *in vitro*. While overall, we observed a general dose-dependent rapamycin-mediated decrease in VEGF expression in both the isogenic cell lines and the parental HS Sultan cell lines, the p27IRES-VEGF transfected cell line consistently maintained greater total levels of VEGF than its isogenic control. These results likely reflect the fact that the mTOR-mediated, cap-dependent VEGF translational pathway was equally sensitive to rapamycin-mediated inhibition in both isogenic cell lines, but that the cap-independent, p27IRES-mediated VEGF salvage pathway was only active in the p27IRES-VEGF transfected cell line. Confirmation that the ectopic VEGF transgene containing the p27IRES expressed VEGF that was resistant to mTOR inhibition was determined by anti-FLAG ELISA, which was used to capture FLAG-tagged VEGF protein (Figure 3(c)). This indicated that the flag-tagged VEGF was expressed in p27IRES-VEGF transfected cells, and that significant levels were maintained even at the highest concentration of rapamycin used. Even though mTOR inhibition did modestly decrease the levels of ectopic VEGF, the levels of VEGF remained significantly greater than what we observed in the control-transfected cell line.

**Figure 4:**
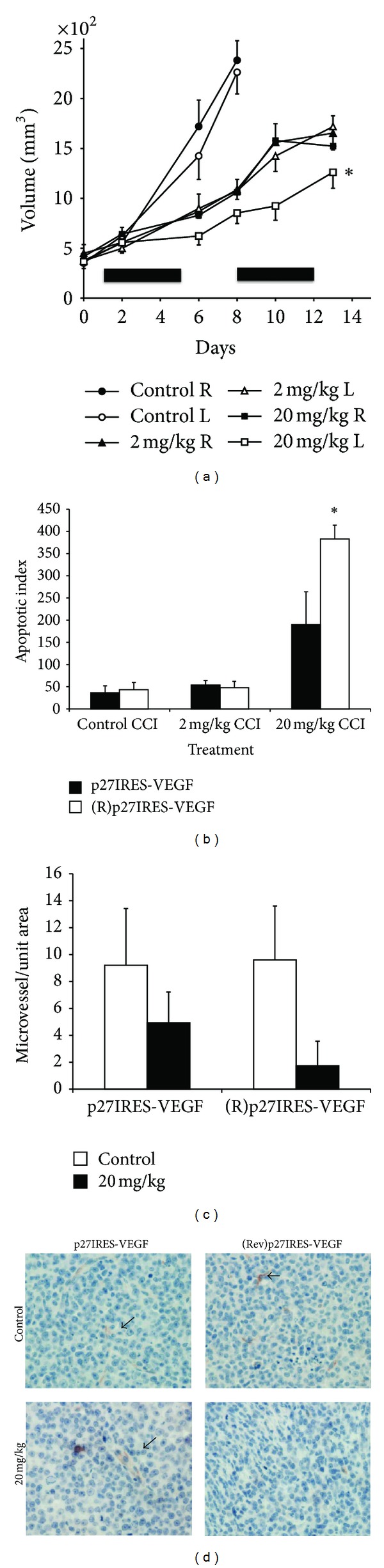
Ectopic VEGF expression rescues tumor cells from the antitumor effects of temsirolimus *in vivo*. (a) NOD/SCID mice (6 mice/group) were challenged subcutaneously with 1 × 10^6^ HS Sultan cells stably transfected with exogenous VEGF expressing p27IRES-VEGF transfected cells (filled symbols) on the right flank and 1 × 10^6^control (Rev)p27IRES-VEGF transfected cells (open symbols) on the left flank. When tumor size reached approximately 500 mm^3^, mice were randomly assigned to receive vehicle alone, 2 mg/kg or 20 mg/kg temsirolimus IP for 10 days, as described in “Materials and Methods.” Results are presented as tumor volume (mean ± SEM). Solid bars on *x*-axis denote days of IP treatment. *Asterisk denotes significant difference* (*P* < 0.05) *between the growth curves for p27IRES-VEGF transfected cells (filled squares) and (Rev)p27IRES-VEGF transfected cells (open squares) in the group of mice treated with 20 mg/kg temsirolimus*. (b) Cleaved caspase-3 staining of isogenic HS Sultan xenografts harvested from mice at day 13 was used to identify apoptosis. Results are presented as number of cleaved caspase-3 stained cells/microscopic field (original magnification 20X), mean ± SD, *n* = 10 fields for each tumor, and 4 tumors/group. Asterisk denotes significant difference (*P* < 0.05) between control and temsirolimus-treated mice. (c) Effect of temsirolimus treatment on the MVD (^#^microvessel/area of microscopic field (original magnification, X20)) stained positive for CD34 and assessed as described in “Materials and Methods.” Data are mean ± SD, *n* = 10 fields/tumor, and 4 tumors/group. Asterisk denotes significant difference (*P* < 0.05) between control and temsirolimus (20 mg/kg) treated mice. (d) Representative slides of CD34-stained isogenic HS Sultan xenograft sections from mice treated with vehicle control (top panels) or 20 mg/kg temsirolimus (bottom panels) harvested on day 13. Original magnification, ×40. Arrow shows microvessel.

We next tested whether ectopic VEGF expressed from the p27IRES-VEGF construct could rescue tumor cells from the antitumor effects of mTOR inhibitors *in vivo*. NOD/SCID mice were challenged subcutaneously with the p27IRES-VEGF transfected cells on the right flank and the control (Rev)p27IRES-VEGF control tumor cells on the left flank. There was no significant difference in the growth of these two isogenic transfected tumor cell lines in the absence of any treatment. Once palpable tumors had formed, the mice were treated with vehicle control, 2 mg/kg or 20 mg/kg temsirolimus as described previously. Both the p27IRES-VEGF and the (Rev)p27IRES-VEGF tumors continued to grow at the same rate in the control treated mice (Figure 4(a), open and closed circles). In fact, a number of the control mice were sacrificed by day 8 because the tumors had become too large and had reached the end-point criteria. However, in the mice treated with high dose temsirolimus (20 mg/kg), the antitumor effect was significantly (*P* < 0.05) decreased when tumors expressed the p27IRES-VEGF construct (compare black versus white squares in Figure 4(a)). In contrast, the more modest antitumor effect at the lower 2 mg/kg dose was equal between the two isogenic tumors (black and white triangles).

After the last injection, some of the mice were sacrificed, and the tumors harvested as described previously. As shown in Figure 4(b), analysis of the apoptotic index in the tumor beds of the xenografts demonstrated that expression of p27IRES-VEGF significantly rescued the tumors from temsirolimus-mediated apoptosis at the highest dose of temsirolimus tested. Consistent with previously shown data (Figure 1(c)), the lower 2 mg/kg dose did not significantly induce apoptosis. It is possible that the modest antitumor effect seen at 2 mg/kg (Figure 4(a)) is solely due to tumor cytostatic effects or alterations in cell size, and these effects are VEGF-independent. To test if the p27IRES-VEGF rescue of apoptosis in high dose (20 mg/kg) treated mice correlated with tumor bed angiogenesis, we stained for CD34+ microvessels. We observed that in control-treated mice, the microvessel density was similar in both isogenic tumor nodules (Figure 4(c)). After treatment with 20 mg/kg temsirolimus, however, a marked suppression of MVD occurred in tumors transfected with the control (Rev)p27IRES-VEGF (91% inhibition, mean of 6 mice/group). In tumors transfected with the p27IRES-VEGF, there was also a temsirolimus-induced decrease in MVD, but this was much less of an effect and did not reach statistical significance (*P* > 0.05). Examples of MVD staining of these tumors are shown in Figure 4(d) (arrows indicate microvessel).

**Figure 5:**
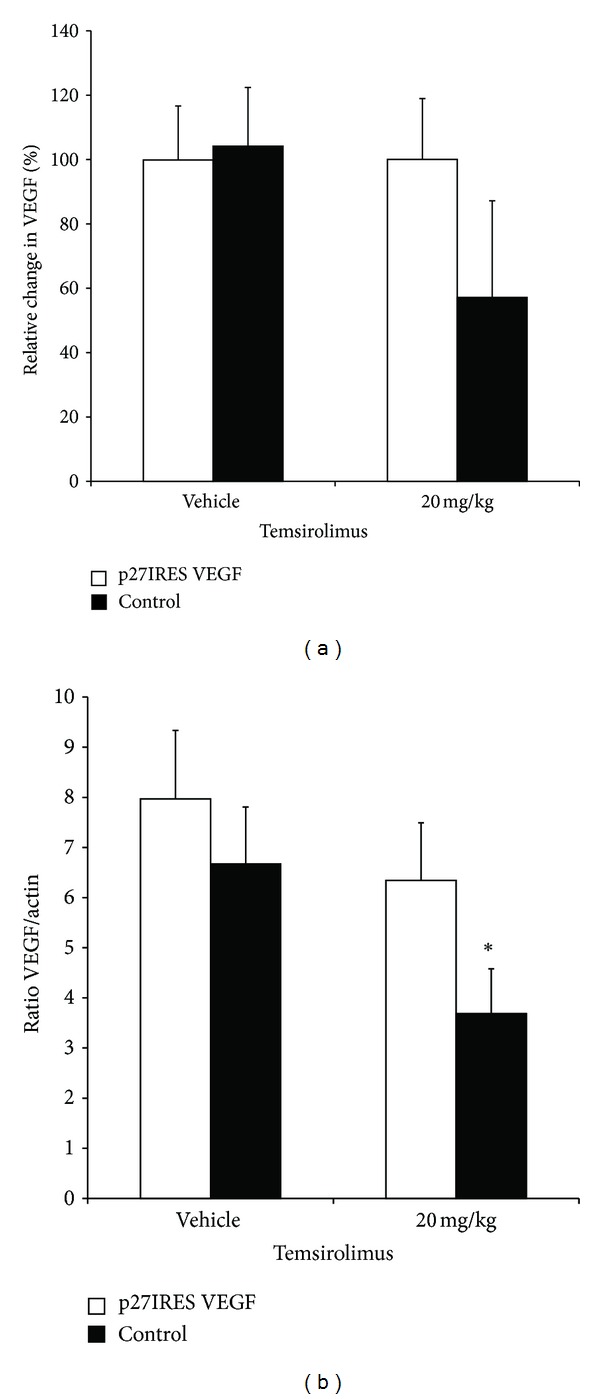
Rescue of VEGF expression in temsirolimus-treated tumors. (a) Relative change in VEGF expression. Isogenic HS Sultan tumors were grown on either flank of NOD/SCID mice (6 mice/group) that were treated with temsirolimus or vehicle control as described in Materials and Methods section. Tumors were harvested, and VEGF levels were measured in the tumor lysates by ELISA. Values are presented as the relative % change of VEGF expression between p27IRES-VEGF (open bars) and (Rev)p27IRES-VEGF (closed bars) tumors isolated from temsirolimus (20 mg/kg) or vehicle control-treated mice. Asterisk denotes significant difference (*P* < 0.05) in relative VEGF expression in (Rev)p27IRES-VEGF transfected cells between control and temsirolimus-treated mice. (b) VEGF expression was also measured in the tumor lysates by immunoblot. VEGF and actin levels were quantified by densitometry analysis and are shown as the ratios of VEGF/actin in p27IRES-VEGF transfected (open bars) and (Rev)p27IRES-VEGF transfected tumors in control or temsirolimus (20 mg/kg) treated mice. Values are the means ± SEM. Asterisk denotes significant difference (*P* < 0.05) in VEGF between the isogenic cell lines in temsirolimus-treated mice.

Next, to confirm that VEGF was differentially expressed in our isogenic xenograft model, VEGF levels were measured in the tumor lysates by ELISA and by immunoblots. First, in a pairwise comparison of isogenic tumors from each mouse, we observed a decrease in VEGF expression (measured by ELISA) in the control (Rev)p27IRES-VEGF tumors cells treated with temsirolimus, but this effect was abrogated in the p27IRES-VEGF transfected tumors (Figure 5(a)). The differences between the groups were just less than statistical significance (*P* < 0.06). We next confirmed that the expression of VEGF was due to expression of the ectopic VEGF transgene by immunoblotting the tumor lysates for FLAG-tagged VEGF using anti-Flag antibodies. Pairwise comparison of the densitometric analysis of the immunoblots (using the ratio of VEGF/actin) of the tumor nodules harvested from 4 different mice demonstrate that the p27IRES VEGF expressing cells maintained significantly higher levels of VEGF expression in the face of mTOR inhibition (*P* < 0.05) (Figure 5(b)), confirming that our transgene was able to maintain ectopic VEGF expression in the p27IRES-VEGF tumors *in vivo*.

### 3.3. VEGF Expression Also Regulates Sensitivity to the Active Site mTOR Inhibitor, pp242

**Figure 6:**
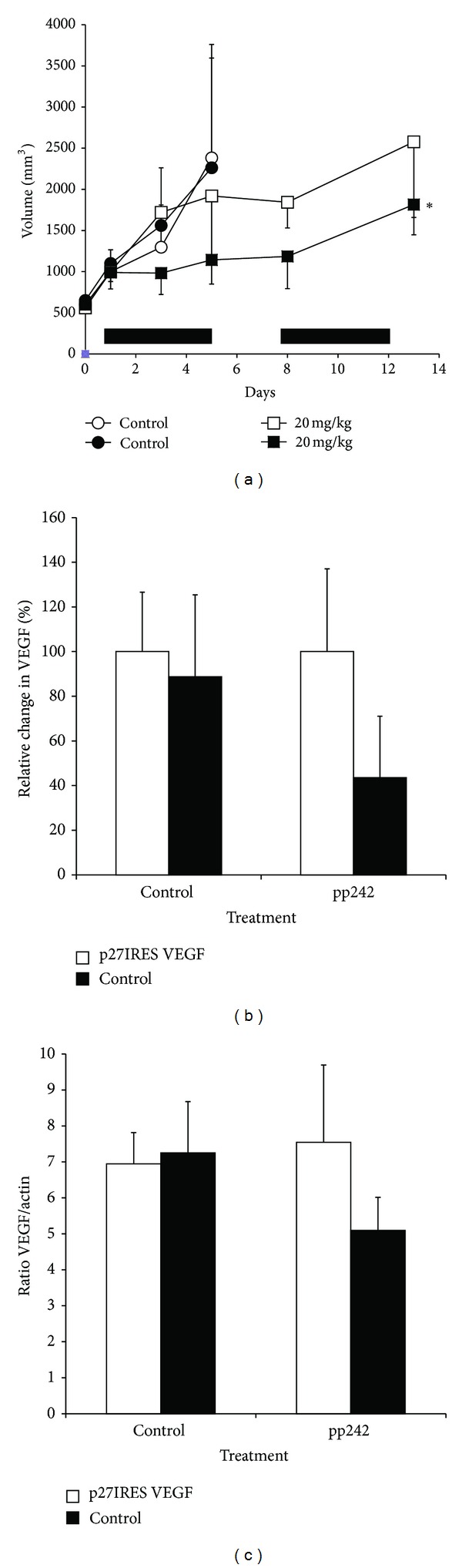
Antitumor effects of the active-site mTOR inhibitor, pp242, are ameliorated by exogenous VEGF expression. (a) NOD/SCID mice (4 mice/group) were challenged subcutaneously with 1 × 10^6^ HS Sultan cells stably transfected with exogenous VEGF expressing p27IRES-VEGF transfected cells (open symbols) on the right flank and 1 × 10^6^control (Rev)p27IRES-VEGF transfected cells (filled symbols) on the left flank. When tumor size reached approximately 500 mm^3^, mice were randomly assigned to receive vehicle alone or 20 mg/kg temsirolimus IP for 10 days, as described in “Materials and Methods.” Mice in the control group were euthanized by day 7 because the tumors had reached end-point criteria. Results are presented as tumor volume (mean ± SEM). Solid bars on *x*-axis denote days of IP treatment. *Asterisk denotes significant difference* (*P* < 0.05) *between the growth curves for p27IRES-VEGF transfected cells (filled squares) and (Rev)p27IRES-VEGF transfected cells (open squares) in the group of mice treated with 20 mg/kg pp242*. (b) Relative change in VEGF expression. Isogenic HS Sultan tumors were grown on either flank of NOD/SCID mice (4 mice/group) that were treated with temsirolimus or vehicle control as described in Materials and Methods section. Tumors were harvested, and VEGF levels were measured in the tumor lysates by ELISA. Values are presented as the relative % change of VEGF expression between p27IRES-VEGF (open bars) and (Rev)p27IRES-VEGF (closed bars) tumors isolated from temsirolimus (20 mg/kg) or vehicle control-treated mice. (c) VEGF expression was also measured in the tumor lysates by immunoblot. VEGF and actin levels were quantified by densitometry analysis and are shown as the ratios of VEGF/actin in p27IRES-VEGF transfected (open bars) and (Rev)p27IRES-VEGF transfected tumors in control or temsirolimus (20 mg/kg) treated mice. Values are the means ± SEM.

New second-generation mTOR inhibitors, so-called “active site inhibitors,” have been developed and are in clinical trials [23, 24]. These agents are more effective than rapalogs in preclinical studies. They inhibit TORC2 as well as TORC1 (rapalogs usually inhibit only TORC1) and are more likely to induce tumor cell apoptosis *in vitro* [25]. However, the *in vivo* mechanisms of action are not clear. A recent study [26] demonstrated a correlation between inhibition of VEGF expression/MVD and an antitumor response in mice treated with an active site mTOR inhibitor but did not directly test if VEGF expression could prevent the response. Therefore, we next tested whether ectopic VEGF expression in our isogenic cell lines could rescue tumor cells from the antitumor effects of the active site mTOR inhibitor, pp242. NOD/SCID mice were challenged SQ as before with both p27IRES-VEGF cells (right flank) or (Rev)p27IRES-VEGF cells (left flank), and once palpable tumors had formed, treatment was initiated with either 20 mg/kg pp242 or DMSO control using the same daily regimen as described previously for the temsirolimus. As seen previously, both the p27IRES-VEGF (open circles) and the (Rev)p27IRES-VEGF (closed circles) tumors grew at the same rate in the absence of treatment. However, in the mice treated with 20 mg/kg pp242, we observed a significant antitumor effect in the (Rev)p27IRES-VEGF control Sultan cells (Figure 6(a), filled squares), whereas the p27IRES-VEGF transfected tumor cells were less sensitivity to the effects of the drug (Figure 6(a), open squares). After the last 5 injections, some of the mice were sacrificed, and the tumors harvested for further analysis as described previously. As shown in Figure 6(b), pp242 downregulated VEGF expression in the control (Rev)p27IRES-VEGF tumor lysates (measured by ELISA), but this effect did not reach statistical significance (*P* < 0.05). Additional analysis demonstrated that VEGF expression was maintained in the p27IRES-VEGF tumors, and that this was due to ectopic VEGF expression which was confirmed by immunoblot analysis FLAG-tagged VEGF (Figure 6(b)) supporting the hypothesis that active site MTOR1/2 inhibitors have similar *in vivo* antitumor effects that are mediated by inhibition of VEGF.

## 4. Discussion

The results of this current study demonstrate that the *in vivo* apoptotic effects of rapalog and active site mTOR inhibitors are caused by the downregulation of VEGF protein expression and the subsequent inhibition of angiogenesis in a B-cell lymphoma xenograft. Histological analysis of the temsirolimus-treated xenografts indicated that tumor cytotoxicity was remarkably colocalized with regions of ischemic stress in the tumor nodules. To prove the role of VEGF inhibition in the cytotoxic effects of mTOR inhibition, we generated isogenic HS Sultan tumor cell lines that expressed a VEGF transgene regulated by the potent p27 IRES sequence [15], thereby allowing us to express ectopic VEGF using the cap-independent salvage pathway. Our *in vivo* data demonstrated that ectopic VEGF rescued tumor from the antitumor effects of two different classes of mTOR inhibitors (temsirolimus and the “active site” inhibitor pp242).

Other studies have suggested that VEGF expression is an important target for mTOR inhibitors. Exposure of tumor cells to these inhibitors *in vitro* results in decreased VEGF expression [9] and, *in vivo*, tumor regression in treated mice frequently correlates with diminished VEGF expression [27] and angiogenesis [28] within tumor beds. To our knowledge, however, the current study is the only one in which ectopic VEGF expression significantly rescued tumors from a cytoreductive and apoptotic response from mTOR inhibitors administered *in vivo*, confirming a critical role for VEGF/angiogenesis rather than the possibility of simple epiphenomena. Although mTOR inhibitor-resistant VEGF expression was maintained by transfection of tumor cells, it is certainly possible that the major apoptotic antitumor effect in our treated mice is due to inhibitory effects of inhibitors on VEGF expression from nonmalignant infiltrating host cells as has been shown in a recent publication [29]. Our data also specifically relate VEGF expression/angiogenesis to an apoptotic effect by mTOR inhibitors. Although use of a lower dose of temsirolimus (2 mg/kg) results in a significant antitumor effect (Figure 4(a)), apoptotic indices are not increased over controls. In addition, this antitumor effect induced by the lower dose is not significantly affected by ectopic VEGF expression (Figure 4(b)). These data indicate a VEGF/angiogenesis-independent antitumor effect is also induced in treated mice that are not associated with tumor cell apoptosis.

We have focused on VEGF translation as a key regulatory event in our model because our prior *in vitro* studies [9] demonstrated that rapalogs primarily inhibited tumor cell VEGF translation rather than RNA levels. However, the situation *in vivo* may be more complicated. As areas of the tumor become hypoxic, a hypoxia inducible factor (HIF) survival response may be initiated but soon aborted due to mTOR inhibition preventing HIF translation and downstream VEGF transcription. Nevertheless, the significant ability of the VEGF transgene to rescue from an apoptotic, antitumor response, only when fused to the p27 IRES demonstrates the importance of cap-independent internal translation in regulating responses to mTOR inhibitors. The VEGF transcript's 5'UTR contains its own IRES structures which appears to become especially important in maintaining VEGF expression during hypoxic stress [30], although this is controversial [31, 32]. However, heightened AKT activity inactivates the VEGF IRES, preventing their function and, thus, allowing sensitization to antitumor effects of mTOR inhibitors [9]. This is particularly true in PTEN-null tumors [12] like the Sultan line exploited here.

It is possible that decreased VEGF/angiogenesis, while required for the *in vivo* apoptotic response, is not sufficient alone and that additional mTOR-inhibitory events occur. For example, the induction of hypoxic stress may initiate the aforementioned HIF survival response *in vivo, *and it is the subsequent inhibition of HIF translation or translation of other antiapoptotic survival proteins that must also occur. Preliminary experiments support this notion. Although exposure of Sultan cells to hypoxia *in vitro* induces modest apoptosis, the addition of rapalogs to the hypoxic environment markedly enhances the apoptotic response, while, as expected, rapalogs themselves in normoxic conditions were ineffective as apoptosis inducers.

## 5. Conclusion

In summary, our data strongly support the idea that mTOR inhibitors induce tumor cell apoptosis *in vivo* by a VEGF/angiogenesis-dependent inhibitory effect. This was even true for mice treated with an active site mTOR inhibitor, pp242, which is reported to induce apoptosis *in vitro* presumably by a direct inhibitory effect on AKT and SGK survival proteins [25]. The data also underscore the importance of cap-independent IRES activity as a key determinant of mTOR inhibitor efficacy.
